# FishTEDB 2.0: an update fish transposable element (TE) database with new functions to facilitate TE research

**DOI:** 10.1093/database/baae044

**Published:** 2024-06-03

**Authors:** Feng Shao, Minzhi Zeng, Xiaofei Xu, Huahao Zhang, Zuogang Peng

**Affiliations:** Key Laboratory of Freshwater Fish Reproduction and Development (Ministry of Education), Southwest University School of Life Sciences, 2 Tiansheng Road, Chongqing 400715, China; Key Laboratory of Freshwater Fish Reproduction and Development (Ministry of Education), Southwest University School of Life Sciences, 2 Tiansheng Road, Chongqing 400715, China; School of Computing Technologies, RMIT University, 124 La Trobe Street, Victoria 3000, Australia; College of Pharmacy and Life Science, Jiujiang University, 551 Qianjin East Road, Jiujiang 332005, China; Key Laboratory of Freshwater Fish Reproduction and Development (Ministry of Education), Southwest University School of Life Sciences, 2 Tiansheng Road, Chongqing 400715, China

## Abstract

We launched the initial version of FishTEDB in 2018, which aimed to establish an open-source, user-friendly, data-rich transposable element (TE) database. Over the past 5 years, FishTEDB 1.0 has gained approximately 10 000 users, accumulating more than 450 000 interactions. With the unveiling of extensive fish genome data and the increasing emphasis on TE research, FishTEDB needs to extend the richness of data and functions. To achieve the above goals, we introduced 33 new fish species to FishTEDB 2.0, encompassing a wide array of fish belonging to 48 orders. To make the updated database more functional, we added a genome browser to visualize the positional relationship between TEs and genes and the estimated TE insertion time in different species. In conclusion, we released a new version of the fish TE database, FishTEDB 2.0, designed to assist researchers in the future study of TE functions and promote the progress of biological theories related to TEs.

**Database URL**: https://www.fishtedb.com/

## Introduction

Transposable elements (TEs) are mobile genomic elements that have long been considered junk sequences (1, 2). However, as research has progressed, studies have found that TEs actually play important roles in organisms, including participating in gene regulation (3), mediating the formation of new genes (4), affecting methylation levels (5), maintaining and shaping 3D genome structure (6), promoting environmental adaptation (7) and participating in species differentiation (8). Moreover, besides their important contributions to organisms, TEs are also important targets for the development of genetic tools, from the early *Sleeping Beauty, PiggyBac* and *Tol2* to *Tn5* used by ATAC-seq technology (9, 10) to newly developed TE genetic manipulation tools (11, 12). The significance of TEs in biological research is increasingly emphasized, with recent research findings on the mechanism of TE transposition garnering significant attention (13, 14), effectively illustrating this point. Abundant TE sequence data will be beneficial for the study of TEs and form the basis of continuous research into biological theories related to TEs.

Fish are an extremely diverse group of vertebrates with early evolutionary origins (15), and therefore, when studying the origin and evolution of vertebrate TEs, this group of animals cannot be ignored. From studies on body color plasticity in animals and fruit phenotype in plants (16–18), we know that TEs can play a role in phenotypic plasticity. The phenotypes of fish are highly plastic (19, 20), and TE–gene interactions can affect these phenotypes, for example, changes in body color and the appearance of egg-spots (21). Thus, we hypothesize that fish might represent a good model for TE–gene–phenotype research. Furthermore, fish TEs have long been popular targets for genetic tool development, such as the widely used *Sleeping Beauty* and *Tol2*, and the newly developed *ZB* (10, 11).

After the first version of FishTEDB was published, FishTEDB 1.0 (22) has gained approximately 10 000 users and has played an important role in research in many fields, including fish genome annotation (23), the evolution of specific superfamily TEs (24) and TE activity and functions (25). Rich TE data offer the opportunity for understanding its evolutionary functions better and developing genetic tools. To this end, the fish TE database (FishTEDB 2.0) was updated to double the number of species and add more functions to the initial version (FishTEDB 1.0) as follows: (i) to promote the study of TE–gene interactions, we have added a function to visualize the positional relationship between genes and TEs. (ii) To promote the development of TE genetic tools, we have added TE insertion time data (recently inserted TEs are more likely to be active (26), which is the basis for genetic manipulation tools). These tools can also be used in a more personalized way according to the needs of the researchers, making it possible to correlate TE insertion time with environmental change (a geological or historical event) to explore the functions of TEs and their associations with genes according to the genomic shock hypothesis (there is a relationship between environmental changes and TE activity) (27). Therefore, the addition of the TE insertion time in FishTEDB will greatly promote the functional research on TEs, and the subsequent addition of more species and updates to the old version of the genome data (FishTEDB 3.0) will further highlight the advantages of FishTEDB.

## Results

### Update with new data

**Figure 1.**
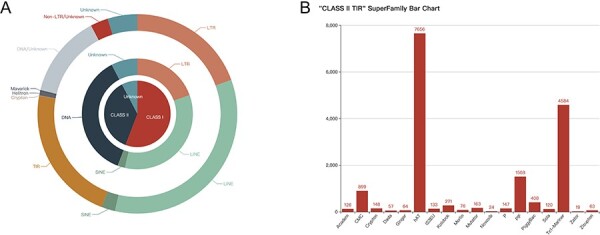
Quantity display of consensus sequences in FishTEDB 2.0. (**A**) Pie chart of classes, types and orders, with the quantity and proportion displayed on the web page. (**B**) Bar chart of different superfamilies in TIR (terminal inverted repeat).

To enhance user convenience, we have not changed the usage or logic of the functions in the initial version of the database. Thus, herein, we do not repeat the usage of FishTEDB 1.0, except for the new features. Within this update, the data volume of FishTEDB 2.0 has significantly increased. Notably, the number of species has increased from 30 to 63 (Supplementary Figure S1), and species coverage has increased from 22 orders and 25 families to 48 orders and 56 families (Supplementary Table S1). The total number of TE sequences has increased from 33 269 to 74 456. Consequently, FishTEDB 2.0 has 2-fold more species and TE sequences than the previous version. More details can be seen in the statistical chart under the ‘Browser’ page (Figure 1).

### Realization of visualization

**Figure 2.**
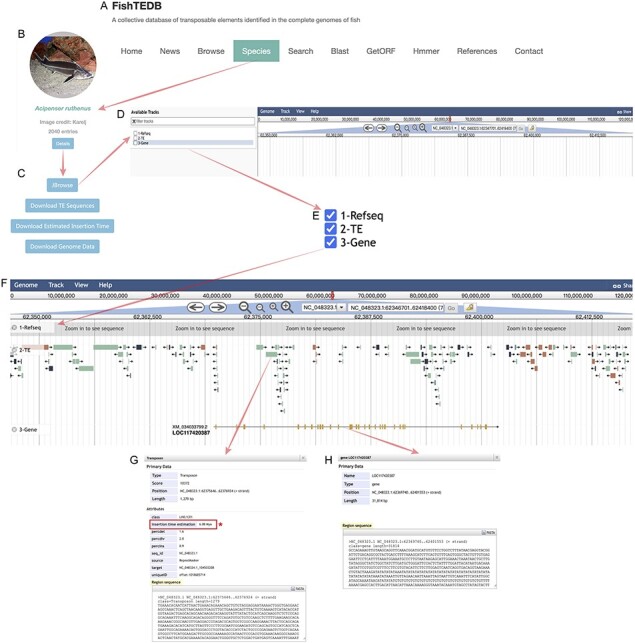
Detailed introduction of new functions in FishTEDB 2.0. (**A**) In the navigation bar of FishTEDB 2.0, the new functions are mainly under the ‘Species’ interface. (**B**) The ‘Details’ button guides the user to a multiple function buttons interface, including the ‘Jbrowse’ button and multiple data download buttons, (**C**) entrance to the visualization of TE distribution, namely, the ‘Jbrowse’ button, (**D**) Jbrowse initial interface and (**E**) information display options in Jbrowse. (**F**) In an example after focusing on a gene, the first track is the sequence (the sequence can be seen at a certain magnification), the second track is the distribution of TEs (colors for different TE types are consistent with colors used in Figure 1 and Supplementary Figure S1), and the third track is the gene. Yellow represents the exon. (**G**) TE-related information, including the location, classification details and sequence information. The red box and asterisk represent the important data update in this version, specifically the insertion time of the TE. (**H**) Gene location, length, sequence and other information.

The most significant enhancement introduced is the integration of visualization features. We visualized the collected gene annotation files of 51 species and TE distribution data of 63 species using JBrowser. The entrance to the corresponding functions was incorporated as a button within the module of the corresponding species under the Species page (Figure 2A); on clicking the ‘Details’ button (Figure 2B) users see multiple function buttons, including the Jbrowse button and multiple data download buttons (Figure 2C). Upon entry to the genome browser (Figure 2C), there are three options on the left side of the entry interface, namely Refseq, TE and Gene (Figure 2D). Researchers are required to check these three items according to their research purpose. Among these, researchers can control the Refseq, TE and gene tracks order according to the check order, and our recommended order is marked with numbers in Figure 2E. If the user wants to observe the structure of a specific gene and its adjacent TEs, it is necessary to zoom in on the corresponding position, which is the same as with the regular usage of JBrowser (28). Different types of TEs are marked with different colors (consistent with the colors used in Figure 1 and Supplementary Figure S1) to make their distribution more intuitive (Figure 2F). Notably, we have not removed the redundancy of the RepeatMasker (http://www.repeatmasker.org) result because it can retain more comprehensive data. This also allows researchers to increase the probability of identifying TE–gene interactions with more alternatives. This will provide detailed information (such as sequence and position information) when the user clicks on a gene or TE. Clicking on a TE opens an information window displaying some results generated by RepeatMasker, such as a series of differences, specifically, percdel (percentage of deletions), percdiv (percentage of divergence) and percins (percentage of insertions) between the sequence and the consensus sequence (Figure 2G). In addition, TE sequences can also be downloaded, which is designed to make TE–gene joint analysis more convenient (Figure 2G). Similarly, clicking on a gene provides its basic information. Users can download the sequence to a local location and then query or compare it in the database for further analysis at a later stage (Figure 2H). As the most important update, the calculated insertion time for a TE is also displayed in a detailed window (Figure 2G), and at the same time, we have added the download function for TE insertion time data. FishTEDB 2.0 is the first TE database to open-source the TE insertion time, which will promote the study of the evolution and function of TEs from the perspective of evolutionary time.

## Discussion

The construction of a TE database can enrich TE data, which is very important for the study of TEs. Fish are an ancient vertebrate group and include many diverse TEs (29, 30); therefore, the construction of a fish TE database will have important roles in tracing the origin of TEs and reconstructing the evolutionary pathways of vertebrate TEs. As RepBase (31) is no longer open source, researchers encounter challenges in obtaining relatively new TE data for free, which makes the establishment of an open-source TE database particularly important and urgent. For FishTEDB, the aim is to continuously update TE data from the fish lineage. Moreover, we participated in the Fish10K Genome Project (32), which will continue to publish large volumes of fish genome data, gradually covering the family level and further the genus level. FishTEDB will also use these genomes to gradually enrich fish TE data, eventually covering the fish genus level.

The TE classification system still needs to be further improved to better understand the evolution of both TE sequences and their host genomes (33). Fish TE superfamilies are rich in diversity (29, 30), coupled with their ancient nature, and they largely cover vertebrate TE superfamilies (except some specialized superfamilies such as the *Alu* superfamily (34), which is unique to primates). Therefore, research on fish TE classification (discovery of new families or superfamilies) might contribute to improvements in the vertebrate TE classification system. FishTEDB 2.0 contains 27 919 TEs that could not be classified into superfamilies, which could represent new TE superfamilies or families that warrant further analyses.

To address the increased amounts of data, the focal point of this update was the implementation of an enriched visual interface. This update could be convenient for advancing TE research. TEs have gradually gained prominence over recent years, and as such, researchers do not only focus on the individual TE itself; instead, TE–gene associations (expression vs repression) have attracted much attention (4). Based on this, our updated version not only introduces the capability of visualizing TE distribution but also integrates gene visualization functionality. This affords users an intuitive depiction of the positional relationships between TEs and genes. Although this function is in its infancy, there are certain limitations in the degree of species richness. However, to some extent, subsequent data updates will transform it into a robust auxiliary tool for fish TE–gene association research.

In this context, the updated inclusion of estimated insertion times for each TE holds more significance because it could have biological meaning, particularly concerning the development of genetic tools and the analysis of environment-specific trait associations across distinct historical periods. Specifically, recently inserted TEs are more likely to be active (26) and could be used as genetic tools. Therefore, identifying recently inserted TEs will greatly improve screening for transpositional efficiency. In addition, according to the genomic shock hypothesis proposed by McClintock (1984), stress and regulatory interference owing to environmental changes in the habitat can lead to the mobilization of TEs (27), and thus, the timing of some environmental changes on the earth, such as the Ice Age, transformations during different geological ages and changes in biological habitats, might affect the behavior of TEs that are related to environmental adaptability in organisms. Therefore, researchers can trace the TE activity at that time to uncover TEs associated with specific traits in a specific historical period or with environmental changes.

## Materials and methods

### Fish genomes data collection and TE prediction

We recollected the genome data of 33 species (in addition to the data of the previous version, FishTEDB 2.0, for a total of 63 species), aiming to cover as many order-level species as possible. At the same time, we collected the gene annotation results of 63 species and downloaded the gene annotation data of 51 species to show the positional relationship between TEs and genes. The prediction of TEs (Supplementary Figure S2) has been described in great detail in our previously published article (25). In this update, we have simplified the de-redundancy step, that is, we have changed from de-redundancy based on the superfamily into DNA, long terminal repeat, long interspersed nuclear element, short interspersed nuclear element and Unknown as units to remove redundancy. The obtained consensus sequence of each species is used as a library, and RepeatMasker (version 4.0.5, http://www.repeatmasker.org/RMDownload.html) is used to identify TEs in the genome of the corresponding species and to obtain the position information of TEs in different species.

### TE insertion time estimation

TE insertion time was obtained using the formula *T* = *K*/2*r* (35), which is widely used for the estimation of TE insertion time (36, 37), wherein *T* represents the insertion time, *K* represents the Kimura distance-based copy divergence of TEs, and *r* represents the nucleic acid substitution rate. To obtain the *K* value, we used the method (https://github.com/4ureliek/Parsing-RepeatMasker-Outputs) developed by Kapusta *et al*. (38) to convert the divergence value in the RepeatMasker result (.out file) to the *K* value. For the process of calculating the *r* value, we used LASTZ (v1.04.00) (http://www.bx.psu.edu/∼rsharris/lastz/), the tools of UCSC utilities (axtChain, ChainNet, netToAxt and axtToMaf; https://github.com/ENCODE-DCC/kentUtils) and MULTIZ (39) with the zebrafish genome as a reference sequence (model species among fishes). For whole-genome alignments, we used the msa_view tool in the PHAST package (40) to extract 4D site alignments based on the zebrafish gene annotations. The phyloFit program in the PHAST package was used to estimate the phylogenetic tree, with a known tree topology as an input parameter, and the tree topology was based on the tree from data presented in our previous study (30), published articles (41) and TIMETREE (http://timetree.org/). The estimated phylogenetic tree is shown in Additional Information 2, and the branch lengths are in units of substitutions per site. We calculated the root-to-tip substitution rates from the latest common ancestor of chordates and vertebrates of each lineage and then divided the root-to-tip substitution rates by the divergence time of the latest common ancestor of chordates and vertebrates (divergence time: 622.6 million years ago) (42), and all *r* value results are shown in Supplementary Table S2.

### Implementation and web interface

The current version of the database, similar to FishTEDB 1.0, was developed using Yii 2.0 (https://github.com/yiisoft/yii2), a high-performance PHP MVC framework for Web 2.0 applications. Various web application development technologies were employed to create the web pages, including Bootstrap 3.3 (https://github.com/twbs/bootstrap), JavaScript (https://tc39.es/ecma262/), jQuery (https://github.com/jquery/jquery) and HTML5 (http://www.w3.org/TR/html5/).

To enhance the scalability of the database, we improved its infrastructure. The entire system was containerized using LXC and deployed on a system image comprising CentOS 7, Nginx 1.14.2 and MySQL 5.7. Subsequently, the image was placed within an LXC cluster, enabling easier expansion to accommodate a larger user base.

We have counted the number of users and determined their global distribution and marked these on the map, located at the bottom of the main page. This was generated using Python with the help of Matplotlib. Access information for the website was extracted from the nginx server log, whereas GeoIP information was obtained from the MaxMind database (https://dev.maxmind.com/geoip/geoip2/geolite2/).

## Supplementary Material

baae044_Supp

## Data Availability

All data are uploaded to FishTEDB 2.0 (https://www.fishtedb.com/) and are open source. The code is available on the Global Ecology Flinders GitHub repository (https://github.com/softflying888/FishTEDB-2.0.git).
