# Supplementary material for: FishTEDB 2.0: an update fish transposable element (TE) database with new functions to facilitate TE research
Source: Database (Oxford). 2024 Jun 3;2024:baae044. doi: 10.1093/database/baae044 (PMC11146639; doi:10.1093/database/baae044)
Supplement: baae044_Supp [file baae044_supp.zip › suppl_data/Additional information 1.docx]

Table S1 Species covered in FishTEDB 2.0 and data sources.

| **Species** | **Order** | **Genome data download links** | **Gene information download links** |
| --- | --- | --- | --- |
| *Acipenser ruthenus* | Acipenseriformes | <https://ftp.ncbi.nlm.nih.gov/genomes/all/GCF/010/645/085/GCF_010645085.2_ASM1064508v2/GCF_010645085.2_ASM1064508v2_genomic.fna.gz> | <https://ftp.ncbi.nlm.nih.gov/genomes/all/GCF/010/645/085/GCF_010645085.2_ASM1064508v2/GCF_010645085.2_ASM1064508v2_genomic.gff.gz> |
| *Amia calva* | Amiiformes | <https://ftp.ncbi.nlm.nih.gov/genomes/all/GCA/016/984/155/GCA_016984155.1_BGI_Acal_1.1/GCA_016984155.1_BGI_Acal_1.1_genomic.fna.gz> | <https://ftp.ncbi.nlm.nih.gov/genomes/all/GCA/016/984/155/GCA_016984155.1_BGI_Acal_1.1/GCA_016984155.1_BGI_Acal_1.1_genomic.gff.gz> |
| *Anguilla anguilla* | Anguilliformes | <https://ftp.ncbi.nlm.nih.gov/genomes/all/GCF/013/347/855/GCF_013347855.1_fAngAng1.pri/GCF_013347855.1_fAngAng1.pri_genomic.fna.gz> | <https://ftp.ncbi.nlm.nih.gov/genomes/all/GCF/013/347/855/GCF_013347855.1_fAngAng1.pri/GCF_013347855.1_fAngAng1.pri_genomic.gff.gz> |
| *Anguilla japonica* | Anguilliformes | <https://ftp.ncbi.nlm.nih.gov/genomes/all/GCA/025/169/545/GCA_025169545.1_ASM2516954v1/GCA_025169545.1_ASM2516954v1_genomic.fna.gz> | None |
| *Antennarius maculatus* | Lophiiformes | <https://ftp.ncbi.nlm.nih.gov/genomes/all/GCA/013/358/685/GCA_013358685.1_fAntMac1.pri/GCA_013358685.1_fAntMac1.pri_genomic.fna.gz> | None |
| *Aplochiton taeniatus* | Galaxiiformes | <https://ftp.ncbi.nlm.nih.gov/genomes/all/GCA/017/639/675/GCA_017639675.1_fAplTae1.pri/GCA_017639675.1_fAplTae1.pri_genomic.fna.gz> | None |
| *Astyanax mexicanus* | Characiformes | <https://ftp.ncbi.nlm.nih.gov/genomes/all/GCF/000/372/685/GCF_000372685.2_Astyanax_mexicanus-2.0/GCF_000372685.2_Astyanax_mexicanus-2.0_genomic.fna.gz> | <https://ftp.ncbi.nlm.nih.gov/genomes/all/GCF/000/372/685/GCF_000372685.2_Astyanax_mexicanus-2.0/GCF_000372685.2_Astyanax_mexicanus-2.0_genomic.gff.gz> |
| *Branchiostoma belcheri* | Amphioxiformes | <https://ftp.ncbi.nlm.nih.gov/genomes/all/GCF/001/625/305/GCF_001625305.1_Haploidv18h27/GCF_001625305.1_Haploidv18h27_genomic.fna.gz> | <https://ftp.ncbi.nlm.nih.gov/genomes/all/GCF/001/625/305/GCF_001625305.1_Haploidv18h27/GCF_001625305.1_Haploidv18h27_genomic.gff.gz> |
| *Callorhinchus milii* | Chimaeriformes | <https://ftp.ncbi.nlm.nih.gov/genomes/all/GCF/018/977/255/GCF_018977255.1_IMCB_Cmil_1.0/GCF_018977255.1_IMCB_Cmil_1.0_genomic.fna.gz> | <https://ftp.ncbi.nlm.nih.gov/genomes/all/GCF/018/977/255/GCF_018977255.1_IMCB_Cmil_1.0/GCF_018977255.1_IMCB_Cmil_1.0_genomic.gff.gz> |
| *Channa argus* | Anabantiformes | <https://ftp.ncbi.nlm.nih.gov/genomes/all/GCA/004/786/185/GCA_004786185.1_ASM478618v1/GCA_004786185.1_ASM478618v1_genomic.fna.gz> | <https://ftp.ncbi.nlm.nih.gov/genomes/all/GCA/004/786/185/GCA_004786185.1_ASM478618v1/GCA_004786185.1_ASM478618v1_genomic.gff.gz> |
| *Chanos chanos* | Gonorynchiformes | <https://ftp.ncbi.nlm.nih.gov/genomes/all/GCF/902/362/185/GCF_902362185.1_fChaCha1.1/GCF_902362185.1_fChaCha1.1_genomic.fna.gz> | <https://ftp.ncbi.nlm.nih.gov/genomes/all/GCF/902/362/185/GCF_902362185.1_fChaCha1.1/GCF_902362185.1_fChaCha1.1_genomic.gff.gz> |
| *Cheilinus undulatus* | Eupercaria/misc | <https://ftp.ncbi.nlm.nih.gov/genomes/all/GCF/018/320/785/GCF_018320785.1_ASM1832078v1/GCF_018320785.1_ASM1832078v1_genomic.fna.gz> | <https://ftp.ncbi.nlm.nih.gov/genomes/all/GCF/018/320/785/GCF_018320785.1_ASM1832078v1/GCF_018320785.1_ASM1832078v1_genomic.gff.gz> |
| *Chelmon rostratus* | Acanthuriformes | <https://ftp.ncbi.nlm.nih.gov/genomes/all/GCF/017/976/325/GCF_017976325.1_fCheRos1.pri/GCF_017976325.1_fCheRos1.pri_genomic.fna.gz> | <https://ftp.ncbi.nlm.nih.gov/genomes/all/GCF/017/976/325/GCF_017976325.1_fCheRos1.pri/GCF_017976325.1_fCheRos1.pri_genomic.gff.gz> |
| *Coilia nasus* | Clupeiformes | <https://ftp.ncbi.nlm.nih.gov/genomes/all/GCA/007/927/625/GCA_007927625.1_knifefish_assembly_2.0/GCA_007927625.1_knifefish_assembly_2.0_genomic.fna.gz> | None |
| *Ctenopharyngodon idellus* | Cypriniformes | <https://ftp.ncbi.nlm.nih.gov/genomes/all/GCF/019/924/925/GCF_019924925.1_HZGC01/GCF_019924925.1_HZGC01_genomic.fna.gz> | <https://ftp.ncbi.nlm.nih.gov/genomes/all/GCF/019/924/925/GCF_019924925.1_HZGC01/GCF_019924925.1_HZGC01_genomic.gff.gz> |
| *Cynoglossus semilaevis* | Pleuronectiformes | <https://ftp.ncbi.nlm.nih.gov/genomes/all/GCF/000/523/025/GCF_000523025.1_Cse_v1.0/GCF_000523025.1_Cse_v1.0_genomic.fna.gz> | <https://ftp.ncbi.nlm.nih.gov/genomes/all/GCF/000/523/025/GCF_000523025.1_Cse_v1.0/GCF_000523025.1_Cse_v1.0_genomic.gff.gz> |
| *Danio rerio* | Cypriniformes | <https://ftp.ncbi.nlm.nih.gov/genomes/all/GCF/000/002/035/GCF_000002035.6_GRCz11/GCF_000002035.6_GRCz11_genomic.fna.gz> | <https://ftp.ncbi.nlm.nih.gov/genomes/all/GCF/000/002/035/GCF_000002035.6_GRCz11/GCF_000002035.6_GRCz11_genomic.gff.gz> |
| *Datnioides undecimradiatus* | Acanthuriformes | <https://ftp.ncbi.nlm.nih.gov/genomes/all/GCA/008/933/995/GCA_008933995.1_BGI_Dund_1.0/GCA_008933995.1_BGI_Dund_1.0_genomic.fna.gz> | None |
| *Dicentrarchus labrax* | Eupercaria/misc | <https://ftp.ncbi.nlm.nih.gov/genomes/all/GCF/905/237/075/GCF_905237075.1_dlabrax2021/GCF_905237075.1_dlabrax2021_genomic.fna.gz> | <https://ftp.ncbi.nlm.nih.gov/genomes/all/GCF/905/237/075/GCF_905237075.1_dlabrax2021/GCF_905237075.1_dlabrax2021_genomic.gff.gz> |
| *Electrophorus electricus* | Gymnotiformes | <https://ftp.ncbi.nlm.nih.gov/genomes/all/GCF/013/358/815/GCF_013358815.1_fEleEle1.pri/GCF_013358815.1_fEleEle1.pri_genomic.fna.gz> | <https://ftp.ncbi.nlm.nih.gov/genomes/all/GCF/013/358/815/GCF_013358815.1_fEleEle1.pri/GCF_013358815.1_fEleEle1.pri_genomic.gff.gz> |
| *Esox lucius* | Esociformes | <https://ftp.ncbi.nlm.nih.gov/genomes/all/GCF/011/004/845/GCF_011004845.1_fEsoLuc1.pri/GCF_011004845.1_fEsoLuc1.pri_genomic.fna.gz> | <https://ftp.ncbi.nlm.nih.gov/genomes/all/GCF/011/004/845/GCF_011004845.1_fEsoLuc1.pri/GCF_011004845.1_fEsoLuc1.pri_genomic.gff.gz> |
| *Gadus morhua* | Gadiformes | <https://ftp.ncbi.nlm.nih.gov/genomes/all/GCF/902/167/405/GCF_902167405.1_gadMor3.0/GCF_902167405.1_gadMor3.0_genomic.fna.gz> | <https://ftp.ncbi.nlm.nih.gov/genomes/all/GCF/902/167/405/GCF_902167405.1_gadMor3.0/GCF_902167405.1_gadMor3.0_genomic.gff.gz> |
| *Gambusia affinis* | Cyprinodontiformes | <https://ftp.cngb.org/pub/gigadb/pub/10.5524/100001_101000/100778/Female_Gambusia_affinis_chromosome.fasta.gz> | <https://ftp.cngb.org/pub/gigadb/pub/10.5524/100001_101000/100778/Female_Gambusia_affinis_gene.gff3.gz> |
| *Gasterosteus aculeatus* | Perciformes | <https://ftp.ncbi.nlm.nih.gov/genomes/all/GCF/016/920/845/GCF_016920845.1_GAculeatus_UGA_version5/GCF_016920845.1_GAculeatus_UGA_version5_genomic.fna.gz> | <https://ftp.ncbi.nlm.nih.gov/genomes/all/GCF/016/920/845/GCF_016920845.1_GAculeatus_UGA_version5/GCF_016920845.1_GAculeatus_UGA_version5_genomic.gff.gz> |
| *Gouania willdenowi* | Gobiesociformes | <https://ftp.ncbi.nlm.nih.gov/genomes/all/GCA/900/650/505/GCA_900650505.1_fGouWil2.1_alternate_haplotype/GCA_900650505.1_fGouWil2.1_alternate_haplotype_genomic.fna.gz> | None |
| *Hemibagrus wyckioides* | Siluriformes | <https://ftp.ncbi.nlm.nih.gov/genomes/all/GCF/019/097/595/GCF_019097595.1_SWU_Hwy_1.0/GCF_019097595.1_SWU_Hwy_1.0_genomic.fna.gz> | <https://ftp.ncbi.nlm.nih.gov/genomes/all/GCF/019/097/595/GCF_019097595.1_SWU_Hwy_1.0/GCF_019097595.1_SWU_Hwy_1.0_genomic.gff.gz> |
| *Hippocampus abdominalis* | Syngnathiformes | <https://ftp.ncbi.nlm.nih.gov/genomes/all/GCA/018/466/805/GCA_018466805.1_ZJU1.0/GCA_018466805.1_ZJU1.0_genomic.fna.gz> | None |
| *Larimichthys crocea* | Eupercaria/misc | <https://ftp.ncbi.nlm.nih.gov/genomes/all/GCF/000/972/845/GCF_000972845.2_L_crocea_2.0/GCF_000972845.2_L_crocea_2.0_genomic.fna.gz> | <https://ftp.ncbi.nlm.nih.gov/genomes/all/GCF/000/972/845/GCF_000972845.2_L_crocea_2.0/GCF_000972845.2_L_crocea_2.0_genomic.gff.gz> |
| *Lateolabrax maculatus* | Acropomatiformes | <https://ftp.ncbi.nlm.nih.gov/genomes/all/GCA/004/023/545/GCA_004023545.1_ASM402354v1/GCA_004023545.1_ASM402354v1_genomic.fna.gz> | None |
| *Lates calcarifer* | Carangaria/misc | <https://ftp.ncbi.nlm.nih.gov/genomes/all/GCF/001/640/805/GCF_001640805.2_TLL_Latcal_v3/GCF_001640805.2_TLL_Latcal_v3_genomic.fna.gz> | <https://ftp.ncbi.nlm.nih.gov/genomes/all/GCF/001/640/805/GCF_001640805.2_TLL_Latcal_v3/GCF_001640805.2_TLL_Latcal_v3_genomic.gff.gz> |
| *Latimeria chalumnae* | Coelacanthiformes | <https://ftp.ncbi.nlm.nih.gov/genomes/all/GCF/000/225/785/GCF_000225785.1_LatCha1/GCF_000225785.1_LatCha1_genomic.fna.gz> | <https://ftp.ncbi.nlm.nih.gov/genomes/all/GCF/000/225/785/GCF_000225785.1_LatCha1/GCF_000225785.1_LatCha1_genomic.gff.gz> |
| *Lepisosteus oculatus* | Lepisosteiformes | <https://ftp.ncbi.nlm.nih.gov/genomes/all/GCF/000/242/695/GCF_000242695.1_LepOcu1/GCF_000242695.1_LepOcu1_genomic.fna.gz> | <https://ftp.ncbi.nlm.nih.gov/genomes/all/GCF/000/242/695/GCF_000242695.1_LepOcu1/GCF_000242695.1_LepOcu1_genomic.gff.gz> |
| *Lucifuga dentata* | Ophidiiformes | <https://ftp.ncbi.nlm.nih.gov/genomes/all/GCA/014/773/175/GCA_014773175.1_Ldentata1.0/GCA_014773175.1_Ldentata1.0_genomic.fna.gz> | <https://ftp.ncbi.nlm.nih.gov/genomes/all/GCA/014/773/175/GCA_014773175.1_Ldentata1.0/GCA_014773175.1_Ldentata1.0_genomic.gff.gz> |
| *Mastacembelus armatus* | Synbranchiformes | <https://ftp.ncbi.nlm.nih.gov/genomes/all/GCF/900/324/485/GCF_900324485.2_fMasArm1.2/GCF_900324485.2_fMasArm1.2_genomic.fna.gz> | <https://ftp.ncbi.nlm.nih.gov/genomes/all/GCF/900/324/485/GCF_900324485.2_fMasArm1.2/GCF_900324485.2_fMasArm1.2_genomic.gff.gz> |
| *Maylandia zebra* | Cichliformes | <https://ftp.ncbi.nlm.nih.gov/genomes/all/GCF/000/238/955/GCF_000238955.4_M_zebra_UMD2a/GCF_000238955.4_M_zebra_UMD2a_genomic.fna.gz> | <https://ftp.ncbi.nlm.nih.gov/genomes/all/GCF/000/238/955/GCF_000238955.4_M_zebra_UMD2a/GCF_000238955.4_M_zebra_UMD2a_genomic.gff.gz> |
| *Megalops cyprinoides* | Elopiformes | <https://ftp.ncbi.nlm.nih.gov/genomes/all/GCF/013/368/585/GCF_013368585.1_fMegCyp1.pri/GCF_013368585.1_fMegCyp1.pri_genomic.fna.gz> | <https://ftp.ncbi.nlm.nih.gov/genomes/all/GCF/013/368/585/GCF_013368585.1_fMegCyp1.pri/GCF_013368585.1_fMegCyp1.pri_genomic.gff.gz> |
| *Myripristis murdjan* | Holocentriformes | <https://ftp.ncbi.nlm.nih.gov/genomes/all/GCF/902/150/065/GCF_902150065.1_fMyrMur1.1/GCF_902150065.1_fMyrMur1.1_genomic.fna.gz> | <https://ftp.ncbi.nlm.nih.gov/genomes/all/GCF/902/150/065/GCF_902150065.1_fMyrMur1.1/GCF_902150065.1_fMyrMur1.1_genomic.gff.gz> |
| *Neolamprologus brichardi* | Cichliformes | <https://ftp.ncbi.nlm.nih.gov/genomes/all/GCF/000/239/395/GCF_000239395.1_NeoBri1.0/GCF_000239395.1_NeoBri1.0_genomic.fna.gz> | <https://ftp.ncbi.nlm.nih.gov/genomes/all/GCF/000/239/395/GCF_000239395.1_NeoBri1.0/GCF_000239395.1_NeoBri1.0_genomic.gff.gz> |
| *Neostethus bicornis* | Atheriniformes | <https://ftp.ncbi.nlm.nih.gov/genomes/all/GCA/902/685/375/GCA_902685375.1_fNeoBic2.1/GCA_902685375.1_fNeoBic2.1_genomic.fna.gz> | None |
| *Nothobranchius furzeri* | Cyprinodontiformes | <https://ftp.ncbi.nlm.nih.gov/genomes/all/GCF/027/789/165/GCF_027789165.1_UI_Nfuz_MZM_1.0/GCF_027789165.1_UI_Nfuz_MZM_1.0_genomic.fna.gz> | <https://ftp.ncbi.nlm.nih.gov/genomes/all/GCF/027/789/165/GCF_027789165.1_UI_Nfuz_MZM_1.0/GCF_027789165.1_UI_Nfuz_MZM_1.0_genomic.gff.gz> |
| *Notothenia coriiceps* | Perciformes | <https://ftp.ncbi.nlm.nih.gov/genomes/all/GCF/000/735/185/GCF_000735185.1_NC01/GCF_000735185.1_NC01_genomic.fna.gz> | <https://ftp.ncbi.nlm.nih.gov/genomes/all/GCF/000/735/185/GCF_000735185.1_NC01/GCF_000735185.1_NC01_genomic.gff.gz> |
| *Oncorhynchus mykiss* | Salmoniformes | <https://ftp.ncbi.nlm.nih.gov/genomes/all/GCF/013/265/735/GCF_013265735.2_USDA_OmykA_1.1/GCF_013265735.2_USDA_OmykA_1.1_genomic.fna.gz> | <https://ftp.ncbi.nlm.nih.gov/genomes/all/GCF/013/265/735/GCF_013265735.2_USDA_OmykA_1.1/GCF_013265735.2_USDA_OmykA_1.1_genomic.gff.gz> |
| *Oreochromis niloticus* | Cichliformes | <https://ftp.ncbi.nlm.nih.gov/genomes/all/GCF/001/858/045/GCF_001858045.2_O_niloticus_UMD_NMBU/GCF_001858045.2_O_niloticus_UMD_NMBU_genomic.fna.gz> | <https://ftp.ncbi.nlm.nih.gov/genomes/all/GCF/001/858/045/GCF_001858045.2_O_niloticus_UMD_NMBU/GCF_001858045.2_O_niloticus_UMD_NMBU_genomic.gff.gz> |
| *Oryzias latipes* | Beloniformes | <https://ftp.ncbi.nlm.nih.gov/genomes/all/GCF/002/234/675/GCF_002234675.1_ASM223467v1/GCF_002234675.1_ASM223467v1_genomic.fna.gz> | <https://ftp.ncbi.nlm.nih.gov/genomes/all/GCF/002/234/675/GCF_002234675.1_ASM223467v1/GCF_002234675.1_ASM223467v1_genomic.gff.gz> |
| *Periophthalmus magnuspinnatus* | Gobiiformes | <https://ftp.ncbi.nlm.nih.gov/genomes/all/GCF/001/515/645/GCF_001515645.1_SAMN03320097.WGS_v1.1/GCF_001515645.1_SAMN03320097.WGS_v1.1_genomic.fna.gz> | <https://ftp.ncbi.nlm.nih.gov/genomes/all/GCF/001/515/645/GCF_001515645.1_SAMN03320097.WGS_v1.1/GCF_001515645.1_SAMN03320097.WGS_v1.1_genomic.gff.gz> |
| *Petromyzon marinus* | Petromyzontiformes | <https://ftp.ncbi.nlm.nih.gov/genomes/all/GCF/010/993/605/GCF_010993605.1_kPetMar1.pri/GCF_010993605.1_kPetMar1.pri_genomic.fna.gz> | <https://ftp.ncbi.nlm.nih.gov/genomes/all/GCF/010/993/605/GCF_010993605.1_kPetMar1.pri/GCF_010993605.1_kPetMar1.pri_genomic.gff.gz> |
| *Planiliza haematocheilus* | Mugiliformes | <https://ftp.ncbi.nlm.nih.gov/genomes/all/GCA/005/024/645/GCA_005024645.1_Liza_haematocheila_0.1/GCA_005024645.1_Liza_haematocheila_0.1_genomic.fna.gz> | None |
| *Poecilia formosa* | Cyprinodontiformes | <https://ftp.ncbi.nlm.nih.gov/genomes/all/GCF/000/485/575/GCF_000485575.1_Poecilia_formosa-5.1.2/GCF_000485575.1_Poecilia_formosa-5.1.2_genomic.fna.gz> | <https://ftp.ncbi.nlm.nih.gov/genomes/all/GCF/000/485/575/GCF_000485575.1_Poecilia_formosa-5.1.2/GCF_000485575.1_Poecilia_formosa-5.1.2_genomic.gff.gz> |
| *Protosalanx chinensis* | Osmeriformes | <https://ftp.ncbi.nlm.nih.gov/genomes/all/GCA/030/340/685/GCA_030340685.1_ASM3034068v1/GCA_030340685.1_ASM3034068v1_genomic.fna.gz> | None |
| *Salarias fasciatus* | Blenniiformes | <https://ftp.ncbi.nlm.nih.gov/genomes/all/GCF/902/148/845/GCF_902148845.1_fSalaFa1.1/GCF_902148845.1_fSalaFa1.1_genomic.fna.gz> | <https://ftp.ncbi.nlm.nih.gov/genomes/all/GCF/902/148/845/GCF_902148845.1_fSalaFa1.1/GCF_902148845.1_fSalaFa1.1_genomic.gff.gz> |
| *Scleropages formosus* | Osteoglossiformes | <https://ftp.ncbi.nlm.nih.gov/genomes/all/GCF/900/964/775/GCF_900964775.1_fSclFor1.1/GCF_900964775.1_fSclFor1.1_genomic.fna.gz> | <https://ftp.ncbi.nlm.nih.gov/genomes/all/GCF/900/964/775/GCF_900964775.1_fSclFor1.1/GCF_900964775.1_fSclFor1.1_genomic.gff.gz> |
| *Siniperca chuatsi* | Centrarchiformes | <https://ftp.ncbi.nlm.nih.gov/genomes/all/GCF/020/085/105/GCF_020085105.1_ASM2008510v1/GCF_020085105.1_ASM2008510v1_genomic.fna.gz> | <https://ftp.ncbi.nlm.nih.gov/genomes/all/GCF/020/085/105/GCF_020085105.1_ASM2008510v1/GCF_020085105.1_ASM2008510v1_genomic.gff.gz> |
| *Sinocyclocheilus graham* | Cypriniformes | <https://ftp.ncbi.nlm.nih.gov/genomes/all/GCF/001/515/645/GCF_001515645.1_SAMN03320097.WGS_v1.1/GCF_001515645.1_SAMN03320097.WGS_v1.1_genomic.fna.gz> | <https://ftp.ncbi.nlm.nih.gov/genomes/all/GCF/001/515/645/GCF_001515645.1_SAMN03320097.WGS_v1.1/GCF_001515645.1_SAMN03320097.WGS_v1.1_genomic.gff.gz> |
| *Sparus aurata* | Eupercaria/misc | <https://ftp.ncbi.nlm.nih.gov/genomes/all/GCF/900/880/675/GCF_900880675.1_fSpaAur1.1/GCF_900880675.1_fSpaAur1.1_genomic.fna.gz> | <https://ftp.ncbi.nlm.nih.gov/genomes/all/GCF/900/880/675/GCF_900880675.1_fSpaAur1.1/GCF_900880675.1_fSpaAur1.1_genomic.gff.gz> |
| *Sphaeramia orbicularis* | Kurtiformes | <https://ftp.ncbi.nlm.nih.gov/genomes/all/GCF/902/148/855/GCF_902148855.1_fSphaOr1.1/GCF_902148855.1_fSphaOr1.1_genomic.fna.gz> | <https://ftp.ncbi.nlm.nih.gov/genomes/all/GCF/902/148/855/GCF_902148855.1_fSphaOr1.1/GCF_902148855.1_fSphaOr1.1_genomic.gff.gz> |
| *Takifugu flavidus* | Tetraodontiformes | <https://ftp.ncbi.nlm.nih.gov/genomes/all/GCF/003/711/565/GCF_003711565.1_ASM371156v2/GCF_003711565.1_ASM371156v2_genomic.fna.gz> | <https://ftp.ncbi.nlm.nih.gov/genomes/all/GCF/003/711/565/GCF_003711565.1_ASM371156v2/GCF_003711565.1_ASM371156v2_genomic.gff.gz> |
| *Takifugu rubripes* | Tetraodontiformes | <https://ftp.ncbi.nlm.nih.gov/genomes/all/GCF/901/000/725/GCF_901000725.2_fTakRub1.2/GCF_901000725.2_fTakRub1.2_genomic.fna.gz> | <https://ftp.ncbi.nlm.nih.gov/genomes/all/GCF/901/000/725/GCF_901000725.2_fTakRub1.2/GCF_901000725.2_fTakRub1.2_genomic.gff.gz> |
| *Tetraodon nigroviridis* | Tetraodontiformes | <https://ftp.ncbi.nlm.nih.gov/genomes/all/GCA/000/180/735/GCA_000180735.1_ASM18073v1/GCA_000180735.1_ASM18073v1_genomic.fna.gz> | <https://ftp.ncbi.nlm.nih.gov/genomes/all/GCA/000/180/735/GCA_000180735.1_ASM18073v1/GCA_000180735.1_ASM18073v1_genomic.gff.gz> |
| *Thalassophryne amazonica* | Batrachoidiformes | <https://ftp.ncbi.nlm.nih.gov/genomes/all/GCF/902/500/255/GCF_902500255.1_fThaAma1.1/GCF_902500255.1_fThaAma1.1_genomic.fna.gz> | <https://ftp.ncbi.nlm.nih.gov/genomes/all/GCF/902/500/255/GCF_902500255.1_fThaAma1.1/GCF_902500255.1_fThaAma1.1_genomic.gff.gz> |
| *Thunnus orientalis* | Scombriformes | From_the_publisher | From_the_publisher |
| *Trachurus trachurus* | Carangiformes | <https://ftp.ncbi.nlm.nih.gov/genomes/all/GCA/905/171/665/GCA_905171665.2_fTraTra1.2/GCA_905171665.2_fTraTra1.2_genomic.fna.gz> | None |
| *Xiphias gladius* | Carangiformes | <https://ftp.ncbi.nlm.nih.gov/genomes/all/GCF/016/859/285/GCF_016859285.1_ASM1685928v1/GCF_016859285.1_ASM1685928v1_genomic.fna.gz> | <https://ftp.ncbi.nlm.nih.gov/genomes/all/GCF/016/859/285/GCF_016859285.1_ASM1685928v1/GCF_016859285.1_ASM1685928v1_genomic.gff.gz> |
| *Xiphophorus maculates* | Cyprinodontiformes | <https://ftp.ncbi.nlm.nih.gov/genomes/all/GCF/002/775/205/GCF_002775205.1_X_maculatus-5.0-male/GCF_002775205.1_X_maculatus-5.0-male_genomic.fna.gz> | <https://ftp.ncbi.nlm.nih.gov/genomes/all/GCF/002/775/205/GCF_002775205.1_X_maculatus-5.0-male/GCF_002775205.1_X_maculatus-5.0-male_genomic.gff.gz> |

Table S2 Species in this study and their substitution rates.

| **Species** | **Branch length** | **r value** |
| --- | --- | --- |
| *Acipenser ruthenus* | 1.038697 | 0.001668322 |
| *Amia calva* | 1.107536 | 0.001778889 |
| *Anguilla anguilla* | 1.298574 | 0.002085728 |
| *Anguilla japonica* | 1.297222 | 0.002083556 |
| *Antennarius maculatus* | 1.835688 | 0.002948423 |
| *Aplochiton taeniatus* | 1.477571 | 0.002373227 |
| *Astyanax mexicanus* | 1.574147 | 0.002528344 |
| *Branchiostoma belcheri* | 0.421177 | 0.000676481 |
| *Callorhinchus milii* | 1.383721 | 0.002222488 |
| *Channa argus* | 1.686385 | 0.002708617 |
| *Chanos chanos* | 1.496985 | 0.002404409 |
| *Cheilinus undulatus* | 1.772588 | 0.002847074 |
| *Chelmon rostratus* | 1.607218 | 0.002581462 |
| *Coilia nasus* | 1.463728 | 0.002350993 |
| *Ctenopharyngodon idella* | 1.551319 | 0.002491678 |
| *Cynoglossus semilaevis* | 2.001397 | 0.003214579 |
| *Danio rerio* | 1.680721 | 0.00269952 |
| *Datnioides undecimradiatus* | 1.664353 | 0.00267323 |
| *Dicentrarchus labrax* | 1.647351 | 0.002645922 |
| *Electrophorus electricus* | 1.573956 | 0.002528037 |
| *Esox lucius* | 1.425431 | 0.002289481 |
| *Gadus morhua* | 1.633285 | 0.00262333 |
| *Gambusia affinis* | 1.987238 | 0.003191837 |
| *Gasterosteus aculeatus* | 1.796644 | 0.002885712 |
| *Gouania willdenowi* | 1.952014 | 0.003135262 |
| *Hemibagrus wyckioides* | 1.607277 | 0.002581556 |
| *Hippocampus abdominalis* | 1.988652 | 0.003194109 |
| *Larimichthys crocea* | 1.665744 | 0.002675464 |
| *Lateolabrax maculatus* | 1.614458 | 0.00259309 |
| *Lates calcarifer* | 1.619641 | 0.002601415 |
| *Latimeria chalumnae* | 1.305589 | 0.002096995 |
| *Lepisosteus oculatus* | 1.190133 | 0.001911553 |
| *Lucifuga dentata* | 1.570278 | 0.00252213 |
| *Mastacembelus armatus* | 1.671397 | 0.002684544 |
| *Maylandia zebra* | 1.75529 | 0.00281929 |
| *Megalops cyprinoides* | 1.216845 | 0.001954457 |
| *Myripristis murdjan* | 1.464361 | 0.002352009 |
| *Neolamprologus brichardi* | 1.763695 | 0.00283279 |
| *Neostethus bicornis* | 1.980704 | 0.003181343 |
| *Nothobranchius furzeri* | 1.890665 | 0.003036725 |
| *Notothenia coriiceps* | 1.737057 | 0.002790005 |
| *Oncorhynchus mykiss* | 1.377348 | 0.002212252 |
| *Oreochromis niloticus* | 1.713619 | 0.002752359 |
| *Oryzias latipes* | 1.996696 | 0.003207029 |
| *Periophthalmus magnuspinnatus* | 2.096359 | 0.003367104 |
| *Petromyzon marinus* | 1.366494 | 0.002194819 |
| *Planiliza haematocheilus* | 1.71307 | 0.002751478 |
| *Poecilia formosa* | 1.935271 | 0.00310837 |
| *Protosalanx chinensis* | 1.489102 | 0.002391748 |
| *Salarias fasciatus* | 1.897002 | 0.003046903 |
| *Scleropages formosus* | 1.354259 | 0.002175167 |
| *Siniperca chuatsi* | 1.54158 | 0.002476036 |
| *Sinocyclocheilus grahami* | 1.541987 | 0.00247669 |
| *Sparus aurata* | 1.647145 | 0.002645591 |
| *Sphaeramia orbicularis* | 1.684965 | 0.002706336 |
| *Takifugu flavidus* | 1.962715 | 0.003152449 |
| *Takifugu rubripes* | 1.939461 | 0.0031151 |
| *Tetraodon nigroviridis* | 2.017706 | 0.003240774 |
| *Thalassophryne amazonica* | 1.68728 | 0.002710055 |
| *Thunnus orientalis* | 1.5726 | 0.002525859 |
| *Trachurus trachurus* | 1.686723 | 0.00270916 |
| *Xiphias gladius* | 1.592323 | 0.002557538 |
| *Xiphophorus maculatus* | 1.933341 | 0.00310527 |

Figure S1. Phylogenetic tree of species in the database and the number of TE types each species. Black species names are species in FishTEDB 1.0, and blue species names are species added in this update. Bars of different colors represent different types of TEs, and the numbers represent the number of corresponding types of TEs.

Figure S2. Pipeline used for TE prediction and the software used in the pipeline.
